# Biological Clocks and Rhythms of Anger and Aggression

**DOI:** 10.3389/fnbeh.2018.00004

**Published:** 2018-01-23

**Authors:** Suzanne Hood, Shimon Amir

**Affiliations:** ^1^Department of Psychology, Bishop’s University, Sherbrooke, QC, Canada; ^2^Department of Psychology, Concordia University, Montreal, QC, Canada

**Keywords:** anger, aggression, circadian rhythm, infradian rhythm, clock genes

## Abstract

The body’s internal timekeeping system is an under-recognized but highly influential force in behaviors and emotions including anger and reactive aggression. Predictable cycles or rhythms in behavior are expressed on several different time scales such as circadian (*circa diem*, or approximately 24-h rhythms) and infradian (exceeding 24 h, such as monthly or seasonal cycles). The circadian timekeeping system underlying rhythmic behaviors in mammals is constituted by a network of clocks distributed throughout the brain and body, the activity of which synchronizes to a central pacemaker, or master clock. Our daily experiences with the external environment including social activity strongly influence the exact timing of this network. In the present review, we examine evidence from a number of species and propose that anger and reactive aggression interact in multiple ways with circadian clocks. Specifically, we argue that: (i) there are predictable rhythms in the expression of aggression and anger; (ii) disruptions of the normal functioning of the circadian system increase the likelihood of aggressive behaviors; and (iii) conversely, chronic expression of anger can disrupt normal rhythmic cycles of physiological activities and create conditions for pathologies such as cardiovascular disease to develop. Taken together, these observations suggest that a comprehensive perspective on anger and reactive aggression must incorporate an understanding of the role of the circadian timing system in these intense affective states.

## Introduction

In the writings of Galen and Aristotle, changes in human tempers were associated with the passage of time, where summer was the season of yellow bile, a humor responsible for a “nature that is angry, insolent, or fierce” (Grant, 2000, p.17). Although humorism has long since been abandoned as a medical perspective, the notion that states of anger and aggressive behavior nonetheless exhibit predictable cycles of waxing and waning across time holds some merit. Essentially all species on Earth possess internal timekeeping mechanisms that govern a multitude of cellular, physiological, and behavioral processes. An abundance of evidence demonstrates that these timekeeping mechanisms form a vital part of our physical and mental health, and that disruptions to their normal functioning can severely compromise emotional state and well-being.

Anger and aggressive behaviors are normal parts of the human behavioral repertoire, and an absence of these can be highly disadvantageous for survival (Green and Phillips, 2004; Waltes et al., 2016). At the same time, there is a maladaptive relationship between excessive anger and health, and this maladaptive relationship implicates the operation of the body’s internal timekeeping mechanisms. Here, we review evidence indicating that there are predictable cycles of anger and aggression in humans and non-human species, and offer critical insight into the mechanisms by which normal operation of the body’s circadian system influences these patterns of aggressive behavior across time. We also examine evidence that demonstrates a complex relationship between excessive anger in humans and disruption of circadian clocks: specifically, that disturbances of the body’s time keeping system increase the likelihood of aggression and irritability; and, reciprocally, that the physiological symptoms of anger and aggression perturb the normal functioning of this system.

## Defining Anger and Aggression

The variety of operational definitions of anger and aggression presents a challenge for establishing whether there are predictable rhythms of these behaviors in humans and non-human species (Kempes et al., 2005; Mathias et al., 2007; Fung et al., 2009). It is important to consider these differences in terminology carefully, because some behaviors and emotional states appear to exhibit predictable rhythms whereas others do not. For example, reactive aggression in humans is argued to differ from proactive aggression, in that the former represents a response to a potential threat and is associated with high arousal and impulsivity, whereas the latter is a low arousal, calculated behavior intended to obtain instrumental ends such as a reward (Hubbard et al., 2002; Kempes et al., 2005). As discussed below, some evidence suggests that humans exhibit predictable cycles in displaying reactive aggression (e.g., Leggett et al., 2015; Hwang et al., 2016), but not proactive aggression. Distinguishing among types of aggressive behaviors is also important for identifying the environmental and physiological mechanisms that underlie each, and for determining how these mechanisms are linked to the internal timekeeping system. For example, in song sparrows, cyclic increases in androgen activity strongly influence territorial aggression in breeding season, but these hormone rhythms do not play the same role in regulating aggressive behavior outside of the breeding season (Wingfield, 2012). For these reasons, we have tried in the following sections to specify the type of aggression under analysis; state the context in which the behavior is displayed; and describe the features of the behavior, where possible.

## The Circadian Oscillatory Network and Biological Rhythms

Endogenous biological clocks regulate patterns of physiological activity and behavior on several time scales. Cycles of change that complete within 24 h are known as *circadian* rhythms and include examples such as the sleep/wake cycle, body temperature change, and release of hormones such as melatonin and cortisol. Circadian rhythms provide an adaptive mechanism for organisms to coordinate physiological functions and behaviors with the predictable 24-h cycle of light and dark on Earth. In mammals, the suprachiasmatic nucleus (SCN) in the hypothalamus contains the master circadian clock, and exposure to daylight provides the dominant cue to synchronize this master clock to the external environment (RW.ERROR—Unable to find reference:249). Other powerful synchronizing cues, or *zeitgebers*, include food consumption and social interaction (Stephan, 2002; Mistlberger and Skene, 2004). Subordinate clocks, or oscillators, also exist throughout the body in tissues such as brain, heart, lungs, liver and endocrine glands (Schibler et al., 2015). By receiving time-of-day information from the SCN via synaptic and diffusible signals, these subordinate clocks coordinate the timing of rhythmic activities throughout the body to the external environment (Mohawk et al., 2012; Dibner and Schibler, 2015).

Although *zeitgebers* are vital for keeping the body’s network of oscillators in time with the 24-h day, they are not sufficient in and of themselves for circadian rhythms to occur. Rather, true clock-controlled functions persist even in the absence of environmental cues (known as “free running” rhythms), and will typically exhibit a period that deviates slightly from 24 h. Furthermore, certain *zeitgebers* such as daylight may be rendered ineffective if the SCN is destroyed. Among the criteria used to establish that a physiological process or behavior is truly under control of an endogenous clock, it must persist under constant (i.e., *zeitgeber*-free) conditions, and be able to synchronize anew (or re-entrain) to the re-introduction of an appropriate *zeitgeber*.

**Figure 1:**
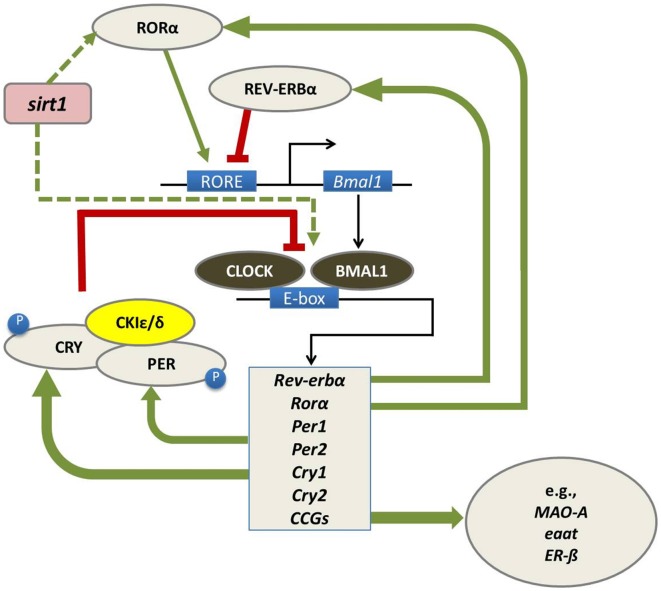
In mammals, the circadian molecular clock comprises a group of genes (“clock genes”) that regulate their own transcription and translation in a cycle that takes approximately 24 h to complete. This regulation is carried out through a series of interlocking negative feedback loops: the transcription factors BMAL1 and CLOCK heterodimerize and promote the expression of the *Period* (*Per1*/*Per2*) and *Cryptochrome* (*Cry1*/*Cry2*) genes, the nuclear receptors retinoid-related orphan receptor (RORα) and REV-ERBα, and a number of downstream genes referred to as clock-controlled genes (CCGs) including monoamine oxidase A (*mao-a*), excitatory amino acid transporter (*eaat*) and estrogen receptor beta (*ERβ*). In turn, the protein products of the *Per* and *Cry* genes dimerize and inhibit the transcriptional activity of CLOCK-BMAL1. The precise timing of this process is regulated by several kinases, such as casein kinase 1 epsilon/delta (CK1ε/δ), which regulate the post-transcriptional activity of PER-CRY dimers. RORα and REV-ERBα also regulate the transcription of BMAL1, whereby RORα promotes its expression, whereas REV-ERBα inhibits it (Huang et al., 2011; Mohawk et al., 2012). Sirtuin-1 (*sirt1*) is believed to regulate this feedback loop at several levels including through promoting RORα-mediated amplification of *bmal* expression (Asher et al., 2008) and acetylation of *bmal* (Nakahata et al., 2008). Figure adapted from Hood and Amir (2017) under a Creative Commons license.

At a molecular level, biological circadian clocks are driven by a core group of genes that regulate their own transcription and translation over 24 h via a series of interacting negative feedback loops (Figure 1; for reviews, see Huang et al., 2011; Mohawk et al., 2012). “Clock” genes regulate their own levels of expression in a predictable cycle that completes in approximately 24 h. Beyond their self-regulation of expression across the day, clock genes play a vital role as transcription factors and control the timing of expression of a wide variety of other genes (referred to as “clock-controlled genes, CCGs”).

Rhythms that complete over time periods exceeding 24 h, such as monthly, seasonally, or annually, are known collectively as *infradian* rhythms. Familiar examples of such rhythms include the menstrual cycle, seasonal breeding and migration behaviors. Although the circadian system is implicated in the expression of infradian rhythms (Oster et al., 2002), the precise mechanisms that regulate these long oscillations remain less clear compared to those underpinning circadian rhythms. A variety of environmental cues regulate the timing of infradian rhythms, depending on the specific rhythm and species under consideration. For example, day length (or photoperiod), ambient temperature and food availability play important roles in the regulation of seasonal breeding rhythms in mammalian and non-mammalian species (for a review, see Paul et al., 2008). These cues exert an impact at a cellular level by initiating changes in melatonin release from the pineal gland, and regulating other endocrine factors such as pituitary hormone signaling (e.g., thyroid, prolactin) and hypothalamic peptides. In turn, these factors ultimately trigger downstream physiological and behavioral changes over time, likely through epigenetic mechanisms (Dawson et al., 2001; Lincoln et al., 2006; Stevenson and Prendergast, 2015; Lynch et al., 2017). In contrast to the presence of a master circadian clock in the SCN, no single tissue appears to house a master infradian timekeeper: rather, evidence to date suggests that several tissues are involved, and that different infradian rhythms engage different networks of tissues (Paul et al., 2008).

## Rhythms of Anger and Aggressive Behavior

### Infradian Cycles

**Figure 2:**
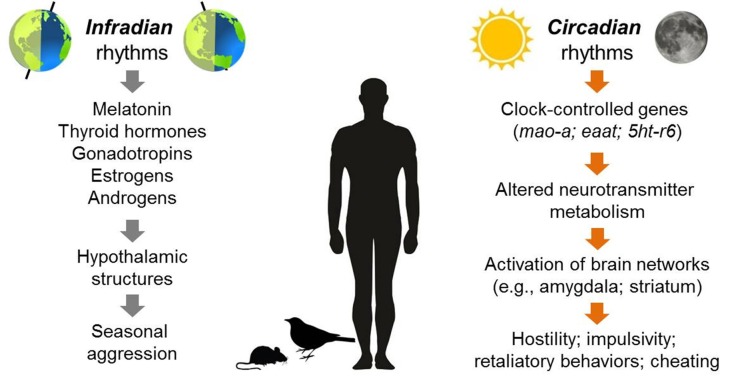
Overview of aggressive behaviors that have been observed to exhibit either seasonal (infradian) or daily (circadian) rhythmicity in humans or non-human species. In terms of infradian rhythms, seasonal changes in daylight length may trigger a variety of hormonal changes that alter the activity of brain structures implicated in aggression such as the ventromedial hypothalamus (VMH). For circadian rhythms, the genetic clockwork that underlies daily cycles of behavior regulates a variety of genes (CCGs, e.g., *mao-a*; *eaat*; serotonin receptors including *5htb)*. In turn, these genes influence the activity of neurotransmitter systems within brain networks involving structures such as the amygdala and striatum. These changes in activation patterns may increase the probability of expressing anger and hostility.

Studies of non-human species provide the strongest evidence for seasonal rhythmicity in the expression of certain types of anger and aggression (for a visual summary of these rhythms, see Figure 2). Aggressive behaviors in many different species of mammals, birds, reptiles, fish and insects exhibit predictable peaks and valleys across the year, and the timing of these behavioral patterns typically exhibits a stable phase relationship with the expression of other seasonal behaviors, such as mating, territory selection, or challenges to social hierarchies (Wilson and Boelkins, 1970; Michael and Zumpe, 1978; Ruby, 1978; Klukowski and Nelson, 1998; Soma, 2006; Perrone et al., 2009; Muschett et al., 2017). For example, some species of non-human primates and ungulates that breed seasonally display more aggression towards others within the social group and sustain more physical injuries from intra-group member attacks (males vs. male) during the few weeks before and at the beginning of sexual partnering. This seasonal rise in aggression has been observed in animals living in laboratory conditions as well as in the wild (Wilson and Boelkins, 1970; Blank et al., 2015). Seasonality in aggressive behaviors is also well-documented in many species of birds and rodents, although the phase relationship of an aggression rhythm with other seasonally fluctuating behaviors such as mating varies. In contrast to the coincident rise of aggression with mating in primates, several species of hamsters and beach mice exhibit more vigorous attack responses towards territory intruders when reproductive activity is low during short photoperiods (Jasnow et al., 2000; Trainor et al., 2007).

The physiological mechanisms that drive seasonal changes in aggression remain only partially understood; however, substantial research in mammals and birds species has identified complex relationships between photoperiod, thyroid hormone levels, and sex hormone levels. In rodents and songbirds, sex hormones play a fundamental role in the regulation of aggression (Ogawa et al., 2000; Sato et al., 2004; Sperry et al., 2010), yet the impact of androgens and estrogens on aggressive behaviors does not remain constant across the seasons. For example, in rodents, estrogens promote aggressive responses to territory intruders when animals are housed in short photoperiod conditions (i.e., winter-like, non-breeding season), but diminish aggression under long photoperiod conditions (Trainor et al., 2007; Laredo et al., 2013). In song sparrows, the role of androgens in modulating aggression also exhibits a seasonal shift, whereby circulating levels of luteinizing hormone and testosterone are necessary for territorial defense behaviors during long photoperiod conditions (the breeding season; Wingfield, 1994, 2012; Sato et al., 2004). Outside of the breeding season, however, displays of territorial aggression are not dependent on these hormones. The triggering of these seasonal differences in behavior appears to rely in part on a photoperiod-mediated endocrine cascade including melatonin and thyroid hormones that drive seasonal fluctuations in sex hormones, although there are significant inter-species differences in this process. For example, the daily period of melatonin release is an important intermediary driver between daylight length, hormone profile and seasonal aggression in rodents (Ono et al., 2008; Rendon et al., 2015), whereas deep brain photoreceptors directly mediate the impact of photoperiod changes, hypothalamic and pituitary hormones, and behavior in songbird species (Nakao et al., 2008; Mukai et al., 2009). Significant work remains to be done to further elucidate these mechanisms. Furthermore, much remains unknown regarding the mechanisms by which non-photoperiod–based seasonal *zeitgebers*, such as food availability, influence circannual cycles of aggressive behavior (Bailey et al., 2016).

In humans, epidemiological evidence indicates that rates of physically aggressive crime fluctuate in phase with seasonal changes in temperature and photoperiod. Analysis of violent crime statistics suggest that events involving personal physical attack (simple and aggravated assault, sexual assault, intimate partner violence) are more likely to occur during the summer season and less likely during the winter, whereas violent crimes not involving direct physical contact (e.g., robbery) do not exhibit seasonal trends (Michael and Zumpe, 1983, 1986; Lauritsen and White, 2014). Other multinational studies of criminally aggressive behavior suggest a similar pattern, whereby rates of physically violent crime (assault, sexual assault) increase during the geographic summer season and decline during the winter (e.g., an increase during July and August in northern nations such as Denmark, and during December and January in southern nations such as Australia); in contrast, non-physical, or property-based crime such as theft showed no seasonal variation (Schreiber et al., 1997). Perhaps surprisingly, however, these studies have not identified similar seasonal cycles in murder rates. Furthermore, global rates of suicide (conceptualized by some as a self-aggressing behavior) also appear to follow a seasonal rhythm, with completed suicides using either violent (e.g., use of firearms, drowning, hanging) or non-violent (e.g., overdose) means increasing slightly in the spring compared to other times of year (Hakko et al., 1998; Woo et al., 2012).

Seasonal trends in physically violent criminal acts have led some to propose a causal relationship between aggressive behavior, temperature and photoperiod (Anderson et al., 2000; Hsiang et al., 2013; discussed in Michael and Zumpe, 1983). Such proposals have not gone without challenge, and alternative interpretations of the relationship include the increased likelihood of social interaction during milder temperatures as compared to periods of cold or extreme heat (Rotton and Cohn, 1999). To date, there is no evidence to suggest that endogenous timekeeping mechanisms are responsible for seasonal changes in violent crime; for example, we are not aware of any demonstration that these patterns free-run in the absence of these environmental cues. Furthermore, there is limited evidence for seasonal variation in human hormone levels or receptor function that is comparable to the mechanisms in non-human species discussed above (Huhtaniemi et al., 1982; Smith et al., 2013).

### Circadian Cycles

Rhythmic patterns in anger and aggressive behavior also have been documented on a circadian time scale (Figure 2). In humans, some evidence suggests that an individual’s *chronotype* (i.e., if one is a morning person or an evening person) is associated with expressions of anger and hostility. Specifically, young to middle-aged adults identifying as evening types tend to score higher on self-report scales of impulsivity, state and trait expressions of anger, and irritability (Park et al., 2015; Hwang et al., 2016; however, see Chrobak et al., 2017), and children and adolescents who are evening types are more likely to be rated by parents or teachers as displaying rule-breaking and externalizing behaviors such as conflict with others, lying, screaming, or swearing compared with individuals who identify as being morning types (reviewed in Schlarb et al., 2014). Interesting experiments also suggest that chronotype may interact with time of day to influence the likelihood of displaying socially transgressive behavior such as cheating: for example, young adults are more likely to lie about their success on monetarily rewarded tasks, like self-reported puzzle solving or dice throwing scores, if they are tested at a time of day that does not coincide with their chronotype (i.e., if morning-type people are tested in the late evening; Gunia et al., 2014; Kouchaki and Smith, 2014; Ingram et al., 2016).

Additionally, there is some evidence for a daily pattern in physically aggressive or agitated motor behaviors and verbal outbursts in individuals suffering from dementia-related disorders such as Alzheimer’s disease. An increase in these behaviors in the late afternoon and early evening has been described as “sundowning” (reviewed in Bachman and Rabins, 2006). Although it is debated in the literature whether sundowning is a real behavioral phenomenon or is instead a methodological artifact (e.g., due to reporting bias in caregivers; Yesavage et al., 2003), some studies indicate that an increase in late-afternoon agitation is likely not attributable to sleep disturbances, which are common in this population (Volicer et al., 2001).

The foregoing examples of daily fluctuations in aggressive behaviors are suggestive of behavioral rhythms; however, these patterns have not yet been shown to meet the criteria of being controlled by an endogenous timekeeper. Nevertheless, several interesting hypotheses could explain the physiological mechanisms by which the circadian system could regulate 24-h susceptibility to anger and aggression. Studies of both human and non-human species indicate that there is a significant genetic component to aggression and anger (for reviews see Takahashi and Miczek, 2014; Waltes et al., 2016), and some of the candidate genes identified to date appear to be clock controlled (Duffield, 2003). For example, individual differences in the activity of several monoamine neurotransmitter systems such as dopamine (DA), norepinephrine (NE) and serotonin (5-HT) are clearly associated with the likelihood of aggressive behaviors in both human and non-human species (Figure 1; Marino et al., 2005; Miczek et al., 2007; Alia-Klein et al., 2008), and some regulatory elements of each of these systems such as catalytic and metabolic enzymes, and receptor proteins are indeed clock controlled (Aston-Jones et al., 2001; Ueda et al., 2002; Weber et al., 2004; Malek et al., 2005). The metabolizing enzyme monoamine oxidase A (MAO-A), which degrades DA, NE and 5-HT, provides a compelling illustration of this. Brain levels of MAO-A are inversely associated with self-reported trait anger in humans (Alia-Klein et al., 2008), and pharmacological blockade of MAO-A activity in rodents increases aggressive responses to intruders (Shih, 2004). The clock proteins BMAL and PER2 positively regulate *mao-a* gene expression in regions of the rodent brain including the striatum and ventral tegmental area, and daily fluctuations in levels of MAO-A in these tissues could presumably influence the likelihood of responding in a more or less aggressive fashion to events happening at different times of day (Hampp et al., 2008). A role for brain glutamatergic and gamma aminobutyric acid (GABA) signaling in aggression has also been demonstrated in non-human species. PER2 positively regulates the expression of the excitatory amino acid transporter (*eaat*) in astrocytes, which contributes to the re-uptake of glutamate from the extracellular space (Abarca et al., 2002; Spanagel et al., 2005; Hampp and Albrecht, 2008; Takahashi and Miczek, 2014).

Clock-controlled regulation of these genes may be particularly important for influencing the activity of brain structures known to mediate the expression of aggression and hostility in mammals. For example, activity in the amygdala is closely linked with aggressive behavior and trait anger, and blunted serotonin activity in this region is associated with elevated aggression and impulsivity in both non-human species and humans (Rosell and Siever, 2015; Suzuki and Lucas, 2015; da Cunha-Bang et al., 2018). Notably, circadian oscillators have been identified within the central and basolateral nuclei of the amygdala (Lamont et al., 2005), and the timing of these oscillators can be shifted by events such as acute glucocorticoid hormone release and exposure to psychological stressors (Segall and Amir, 2010; Al-Safadi et al., 2015). Such events are also known to increase displays of reactive aggression (Mikics et al., 2007).

The genetic circadian clock also plays an important role in mediating the activity of signaling systems such as sex hormones in the ventromedial hypothalamus (VMH), a region strongly implicated in the expression of aggressive behaviors (Cai et al., 2008; Falkner and Lin, 2014). A number of studies demonstrate that changes in the expression of sex hormone receptors in the VMH and in the firing rates of neurons in this region contribute to the display of aggressive behaviors by rodents and songbirds (Spiteri et al., 2010; Lee et al., 2014; Falkner et al., 2016; Wacker et al., 2016). Evidence suggests that the VMH houses a circadian oscillator that is under control of signals from the SCN master clock (Inouye, 1983; Egawa et al., 1993; Ono et al., 1987).

More recent evidence has implicated the role of the nicotinamide adenine dinucleotide-dependent sirtuin proteins (SIRT)—specifically SIRT1—in both the regulation of the genetic circadian clock and the risk for diagnosis of antisocial personality disorder in humans (Chang et al., 2017). SIRT1 regulates the expression of *bmal1* and *per2* through deacetylation (Asher et al., 2008; Nakahata et al., 2008), and overexpression of *sirt1* in rodents leads to elevated levels of *bmal1* and *per2*, and shortens the period of the circadian clocks in the brain controlling locomotor activity (Chang and Guarente, 2013). In a sample of young men, some of whom were juvenile offenders and had received a diagnosis of antisocial personality disorder, a particular single nucleotide polymorphism (SNP) in the *sirt1* gene was associated with a lower risk of antisocial personality disorder diagnosis, whereas a second SNP was found to be more frequent among youth who had received a diagnosis. Although these associations were modest, these observations, when combined with additional evidence linking SIRT1 to the development of midbrain dopaminergic activity, raise interesting questions regarding a link between SIRT1, the circadian clock, monoamine neurotransmitter systems and aggressive behaviors (Lee et al., 2009; Kishi et al., 2011).

Individual genetic differences leading to chronotype may also underlie the association of daily fluctuations in anger and aggression and the circadian timekeeping system. Recent genome-wide association studies in humans have identified multiple loci associated with a morningness type, and many of these loci are near clock genes or genes implicated in the phototransduction process mediating the transfer of daylight information to neurons in the SCN. Interestingly, loci were also identified near genes implicated in the regulation of serotonin activity (*5htr6*) as well as GABAergic activity in brain (*plcl1*; *nol4*), suggesting that the genetic profile that influences morningness or eveningness preference could also involve differences in the activation or sensitivity of these neurotransmitter systems (Hu et al., 2016; Jones et al., 2016). At a behavioral level, chronotype influences changes in cognitive alertness across the day, whereby performances on tests of memory function, reaction time and decision making are worse if one is tested at a time of day that does not align with self-reported morningness/eveningness preference or phase of the genetic circadian clock (May, 1999; Ingram et al., 2016). This worsening of cognitive performance outside of one’s self-identified “optimal” time may increase the likelihood of poorer decision making and impulsiveness, and in turn predispose the expression of anger or hostility in the face of challenging or frustrating circumstances.

## Disruption of the Circadian System and Aggression

In addition to the forgoing evidence suggesting a role for biological clocks in regulating anger and aggressive behaviors, a significant body of research suggests that disruptions of normal biological rhythms also influence these behaviors. The exact nature of this relation remains unclear, as much of the evidence collected on this topic to date in human populations is correlational. As such, it is an open question as to whether one particular direction of relationship is more influential than the other—that is, whether disruptions of rhythmic behaviors promote aggression, or whether heightened arousal and anger disrupt biological clocks, or both (Kamphuis et al., 2012). Below, we examine the evidence that disruptions of circadian rhythms such as the sleep/wake cycle do in fact modulate the expression of anger and hostility in several species. The inverse relation—whether intense or chronic expressions of anger and aggression can impact the functioning of the circadian system itself—will be discussed in the final section of this review.

A common example of a perturbation of the circadian system is the disruption of the normal sleep/wake cycle. Although sleep is not exclusively governed by biological clocks, even short-term periods of sleep deprivation can negatively affect a number of other physiological and behavioral rhythms, and transiently alter the genetic clock in a variety of tissues (for review, see Archer and Oster, 2015; Cedernaes et al., 2015; Gil-Lozano et al., 2016). A number of studies have explored the link between the disruption of sleep and the expression of aggressive behavior. To date, this literature has revealed a complex relationship, in that the impact of sleep impairment on aggression varies depending on the species and the nature of the aggressive behavior under consideration (for review see Kamphuis et al., 2012). In humans, poor sleep quality and loss of sleep correlate with higher self-reported ratings of irritability and experience of anger, and, in the case of studies of children and adolescents, greater instance of aggressive physical activity and hyperactivity based on observer ratings (Waters et al., 1993; Gregory et al., 2004; Grano et al., 2008; Coulombe et al., 2011). Interventions to improve sleep quality appear to lessen these behaviors in some cases (Haynes et al., 2006; Mitchell and Kelly, 2006). It has been noted that many of the studies investigating the relation between sleep loss and aggression in adults have used self-report measures of irritability or responses to hypothetical social scenarios as a measure of aggression, rather than objective assessments of external behavior. However, an interesting exception to this includes observations of on-the-job behavior in shift workers. For example, a large-scale study of police officers in North America revealed that approximately 40% experienced some form of chronic sleep disorder such as apnea or insomnia, and that affected individuals were more likely to have demonstrated adverse work-related behavior such as displaying uncontrolled anger towards suspects or citizens (Rajaratnam et al., 2011). Studies in animal models, particularly rodents, are consistent with the view that sleep deprivation is associated with an increased likelihood of physically aggressive behaviors (Licklider and Bunch, 1946; Webb, 1962; Hicks et al., 1979).

Complicating this picture of the relation between sleep and aggression are several studies that have failed to demonstrate an impact of sleep disruption on aggression, and still others that suggest sleep deprivation may actually diminish aggressive behaviors. For example, in humans, an acute period of sleep deprivation (33 h) decreased the likelihood in male participants of displaying retaliatory behavior towards an opponent in a computer game (Cote et al., 2013). In *Drosophila*, a 12-h period of sleep deprivation similarly reduced physically aggressive behaviors towards other males, and following a sleep recovery period these behaviors were restored (Kayser et al., 2015). Given these differences in the literature, it is difficult to determine conclusively how sleep interacts with aggression, although there is clearly some kind of modulatory influence.

Several mechanisms by which sleep disruption might drive the likelihood of aggressive behavior have been proposed. Sleep loss is known to negatively impact both simple and more complex aspects of cognitive performance including executive function skills and inhibitory regulation (as reviewed in Killgore, 2010). Sleep loss also worsens mood state; increases sensitivity to negatively valenced emotional stimuli; impairs the accuracy of judgment of emotion expressed by human faces; and is associated with poorer inhibitory emotion regulation and decreased empathy towards others (Pilcher and Huffcutt, 1996; Yoo et al., 2007; Killgore et al., 2008; Hisler and Krizan, 2017). This pattern of behavioral change has been linked to impaired functioning of prefrontal cortex, and indeed metabolic activity in this brain region has been observed to decrease following a brief period of sleep deprivation (Harrison and Horne, 2000; Drummond and Brown, 2001). Together, a reduction in executive control, emotion regulation, and weakened perception of emotional states in others could create conditions whereby sleep-deprived individuals are at greater risk of displaying poorly controlled emotional reactions in situations of interpersonal conflict.

Mutations of the genetic clock itself in animal models have also been documented to increase the likelihood of aggression and associated behaviors such as hyperactivity and impulsivity. For example, mice with a knockout of the clock gene *rev-erb alpha* display more aggression towards a territory intruder compared to wild type mice, as well as exhibit greater locomotor activity and exploration behavior (Chung et al., 2014; Jager et al., 2014). The *clock delta 19* mutant mouse, which lacks a CLOCK protein capable of transcription regulation, also exhibits hyperactivity and impulsivity (Roybal et al., 2007; Coque et al., 2011). Associated with the behavioral features of both of these models appears to be the dysregulation of midbrain DA systems, whereby the corresponding gene mutation has been found to create a hyperdopaminergic state through the disinhibition of DA synthesis in midbrain cell groups (in the case of the *rev-erb alpha* KO model) or hyperexcitability of midbrain DA neuron firing (in the case of the *clock delta 19* mutant). Correspondingly, manipulations that diminish this hyperdopaminergic activity appear to attenuate these aggressive phenotypes. Catecholamine dysregulation also appears to be implicated in the effects of sleep deprivation on aggression in *drosophila*, whereby the administration of an octopamine agonist in sleep deprived flies restores the display of physically aggressive behaviors towards another male (octopamine in insects is akin to NE in mammals; Kayser et al., 2015). Taken together, these findings suggest that the dysregulation of midbrain dopaminergic systems by an atypically functioning clock may predispose aggressive and hyperactive behaviors in these models.

## Impact of Anger and Aggression on Endogenous Clocks

Because the circadian network is a physiological system that is exquisitely sensitive to feedback signals arising from both within the body and outside it, it is perhaps not surprising that intense emotional states themselves have been found to influence the pattern of several rhythms acutely. For example, during the night after an episode of work-related or interpersonal conflict, individuals report poorer sleep quality and more sleep disruptions. The strength of this effect appears to be mediated by the extent to which individuals exhibit cynical hostility (a personality construct characterized by negative attitudes towards others, mistrust and defensiveness) and their tendency to ruminate on distressing events (Brissette and Cohen, 2002; Radstaak et al., 2014). In chronically stressed individuals, previous day experiences of anger have been found to predict a blunted amplitude of the cortisol rhythm on the following day (Leggett et al., 2015; Oberle et al., 2017; although see Adam et al., 2006). Acute, anger-provoking stressors have also been observed to blunt the diurnal rhythm of reactive oxygen species production, often used as a marker of immune system activity, in young adults completing a computerized visual judgment task (Atanackovic et al., 2003). Additional evidence suggests that personality disposition towards anger and hostility may perturb biological rhythms over longer time scales. Higher trait anger is predictive of greater sleep fragmentation, poorer self-reported sleep quality, and increased risk for sleep disorders such as insomnia (Waters et al., 1993; Grano et al., 2008; Taylor et al., 2013; Hisler and Krizan, 2017).

The experience of anger is often characterized by physiological symptoms of intense autonomic nervous system activation including transient elevations in blood pressure, heart rate, and sympathetic neurochemical tone, and changes in each of these activities have been found to influence the timing of biological rhythms and clock gene expression in several respects (for a review, see Buijs et al., 2016; Sasaki et al., 2016). For example, substantial research in cell cultures and animal models has demonstrated convincingly that acute surges in corticosteroids can act as a zeitgeber for tissue-specific clocks such as within the limbic system of the brain, liver, kidney and heart (Balsalobre et al., 2000; Al-Safadi et al., 2015). As such, it is possible that frequent and/or intense spikes in sympathetic activation in highly emotional or aggressive individuals may create “noisy” internal feedback conditions that impair the daily entrainment of biological clocks to other important signals.

It should be noted that the effect sizes reported in several of the studies of humans discussed above are modest; nevertheless, even small changes in rhythms such as sleep, when accrued across time, may exert a negative effect on physical health and cognitive performance (Vgontzas et al., 2004; Wang et al., 2016). Given the delicate relationship between biological rhythms and vital physiological functions, anger-induced disruptions of the biological clock may be a key mechanism underlying the increased risk of high trait-anger individuals to physical illness such as cardiovascular disease and inflammatory disorders (Gouin et al., 2008; Haukkala et al., 2010; Boylan and Ryff, 2013). Individuals predisposed towards trait anger or aggression exhibit larger, more prolonged changes in heart rate, galvanic skin responses and blood pressure to stressful events (for review, see Waters et al., 1993; Smith et al., 2004), and this exaggerated reactivity may underlie the attenuated nighttime declines in heart rate and blood pressure observed in these individuals (Linden et al., 2008; Beatty and Matthews, 2009; Pavek and Taube, 2009). Given the evidence that chronic variability in blood pressure and heart rate are risk factors for cardiovascular disease (Kikuya et al., 2000), it is possible that anger-induced perturbation in the biological rhythms of vascular activity may be an important contributor towards exacerbating this risk. If so, the circadian system could represent an important target for therapeutic interventions intended to reduce the risk of illness in individuals who have poorly controlled anger or hostility.

## Conclusion

Taken together, the findings reviewed here demonstrate a meaningful role for biological clocks in anger and aggression. Seasonal changes in the behaviors of several non-human species provide some of the most convincing demonstrations of predictable cycles in the expression of certain types of aggression. It is less clear that true, clock-controlled seasonal cycles exist in human aggression; however, stronger evidence suggests that circadian patterns are present, and that clock-related individual differences such as chronotype are associated with the propensity for anger and aggression. There are a number of hypothesized mechanisms that could account for how the biological timekeeping system at a genetic level influences the cellular and physiological factors that regulate aggressive behaviors, and further research into the role of clock genes in controlling catecholaminergic midbrain systems may be particularly useful in understanding these mechanisms more deeply. Further studies at this level of analysis may also provide more definitive insight into how disruptions of endogenous clocks increase the likelihood of hostile behaviors. Advancements in our understanding of the circadian system’s role in aggression and anger may perhaps be most valuable for improving our ability to prevent and treat the health complications that individuals high in trait anger are at higher risk of developing.

## Author Contributions

SH and SA wrote the manuscript.

## Conflict of Interest Statement

The authors declare that the research was conducted in the absence of any commercial or financial relationships that could be construed as a potential conflict of interest.
